# Microbiological Spectrum and Antimicrobial Resistance Patterns in Tubo‐Ovarian Abscesses Managed by Surgery or Drainage at a Tertiary Hospital in Vietnam: A Retrospective Case Series

**DOI:** 10.1002/hsr2.73414

**Published:** 2026-10-26

**Authors:** Bui Chi Thuong, Huynh Tan An, Pham Dinh Nguyen

**Affiliations:** ^1^ University of Medicine and Pharmacy Ho Chi Minh City Vietnam; ^2^ Nhan Dan Gia Dinh Hospital Ho Chi Minh City Vietnam; ^3^ Thu Duc Hospital Ho Chi Minh City Vietnam; ^4^ Children's Hospital 1 Ho Chi Minh City Vietnam

**Keywords:** antimicrobial resistance, case series, Escherichia coli, gynecologic surgery, tubo‐ovarian abscess, Vietnam

## Abstract

**Background and Aims:**

Tubo‐ovarian abscess requires empirical antimicrobial therapy before culture results are available, but local antimicrobial resistance data are limited in Vietnam. We described the microbiological spectrum and antimicrobial susceptibility patterns of tubo‐ovarian abscesses managed by surgery or drainage.

**Methods:**

We conducted a single‐center retrospective case series of 66 patients with tubo‐ovarian abscess who underwent surgery or drainage and had procedure‐obtained abscess material submitted for culture at Nhan Dan Gia Dinh Hospital from January 2021 to May 2025. Antimicrobial susceptibility data were summarized for recovered isolates when available. Descriptive statistics and Wilson 95% confidence intervals were used for key proportions. Antimicrobial susceptibility was summarized using the number of isolates tested for each agent as the denominator.

**Results:**

The analytic sample comprised 66 surgically treated or drained patients. Culture positivity was 43.9% (29/66; 95% CI, 32.6%–55.9%), with 39 bacterial isolates reported. Escherichia coli was the most frequent isolate (19/39, 48.7%), followed by Streptococcus spp. (9/39, 23.1%) and Klebsiella pneumoniae (7/39, 17.9%). Among E. coli isolates, 6/19 (31.6%) were extended‐spectrum beta‐lactamase producers and 1/19 (5.3%) was an AmpC producer. Resistance among tested E. coli isolates was 13/14 (92.9%) to ampicillin, 7/14 (50.0%) to ceftriaxone, and 10/19 (52.6%) to trimethoprim‐sulfamethoxazole. Antibiotic‐specific denominators were small and variable.

**Conclusion:**

Tubo‐ovarian abscesses managed by surgery or drainage in this Vietnamese case series frequently yielded enteric Gram‐negative organisms and streptococci, with substantial *E. coli* resistance to commonly used agents among tested isolates. These findings support procedure‐obtained abscess culture, local antibiograms, clinically indicated anaerobic coverage, and antimicrobial stewardship; they should not be generalized to all patients with tubo‐ovarian abscess.

## Key Points

**What is known:** Tubo‐ovarian abscess is a polymicrobial pelvic infection requiring empirical broad‐spectrum therapy, but local antimicrobial susceptibility data from Vietnam remain limited.

**What is new:** In this surgically treated or drained case series, *E. coli* was the leading recovered isolate, and resistance to ampicillin, ceftriaxone, and trimethoprim‐sulfamethoxazole was frequent among tested *E. coli* isolates.

**Clinical implications:** Culture of abscess material obtained during surgery or drainage and locally updated antibiograms can support culture‐guided stewardship; small and incomplete AST denominators do not justify automatic broad‐spectrum escalation.

**Abbreviations**
AMR: antimicrobial resistance
AST: antimicrobial susceptibility testing
CI: confidence interval
CLSI: Clinical and Laboratory Standards Institute
CRP: C‐reactive protein
CT: computed tomography
ESBL: extended‐spectrum beta‐lactamase
MDR: multidrug‐resistant
PID: pelvic inflammatory disease
TOA: tubo‐ovarian abscess
WBC: white blood cell.

## 1 Introduction

Pelvic inflammatory disease is an infection of the upper female genital tract and may progress to tubo‐ovarian abscess, a destructive inflammatory mass involving the fallopian tube, ovary, and adjacent pelvic structures [1, 2]. Tubo‐ovarian abscess is clinically important because delayed recognition or ineffective antimicrobial therapy may lead to rupture, peritonitis, sepsis, prolonged hospitalization, and reproductive morbidity [1, 3]. Contemporary management combines broad empirical antimicrobial therapy with image‐guided drainage or surgery when there is clinical deterioration, suspected rupture, or failure of medical therapy [1, 4, 5]. Pelvic inflammatory disease remains an important cause of morbidity, although its measured burden varies by population and surveillance method [6].

Antimicrobial selection for tubo‐ovarian abscess is challenging because the infection is often polymicrobial and culture results may be delayed or negative; prior antimicrobial exposure is a plausible contributor to culture negativity [7]. Recommended pelvic inflammatory disease regimens provide coverage for sexually transmitted pathogens, enteric Gram‐negative organisms, streptococci, and anaerobes, and anaerobic coverage is emphasized when a tubo‐ovarian abscess is present [1, 4]. Emerging resistance among Enterobacterales, including extended‐spectrum beta‐lactamase‐producing Escherichia coli, can reduce the reliability of cephalosporin‐based empirical therapy in some settings [8, 9, 10].

Local microbiology is therefore central to antimicrobial stewardship. Resistance prevalence and the organisms recovered from tubo‐ovarian abscesses vary across settings, making direct extrapolation from international guidance potentially unreliable [7, 8, 9, 10]. Recent prospective evidence also suggests that classic sexually transmitted pathogens may account for only a subset of pelvic inflammatory disease and tubo‐ovarian abscess microbiology in some contemporary populations [11]. Data describing surgically treated tubo‐ovarian abscesses in Vietnamese tertiary hospitals remain limited.

Accordingly, this study aimed to describe the microbiological spectrum and antimicrobial susceptibility patterns in tubo‐ovarian abscesses managed by surgery or drainage at a tertiary hospital in Vietnam. Multidrug‐resistance phenotypes recorded in the available microbiology data were summarized descriptively and interpreted considering incomplete routine AST panels.

## 2 Methods

### 2.1 Study Design and Setting

This single‐center retrospective case series was conducted at the Department of Gynecology, Nhan Dan Gia Dinh Hospital, Ho Chi Minh City, Vietnam. The retrospective record interval reported for this analysis was January 2021 to May 2025. The study included 66 records. A patient‐level sampling frame and screening log were unavailable; therefore, total sampling could not be established. The study was not registered before manuscript submission, and no separate publicly accessible protocol was available. Reporting was cross‐checked against the PROCESS 2023 guideline for surgical case series [12] and the STROBE framework for observational studies [13]. The completed STROBE and PROCESS 2023 checklists are provided as Supporting Information S1 and S2, respectively.

### 2.2 Participants and Case Selection

The analytic sample comprised 66 medical records of patients diagnosed with tubo‐ovarian abscess who underwent surgery or drainage and had procedure‐obtained abscess material submitted for culture during the study period. Antimicrobial susceptibility data were summarized for recovered isolates when available. A complete screening log was unavailable; therefore, the number assessed for eligibility, exclusion counts, and total‐sampling status could not be determined. Culture status was classified using the recorded monomicrobial and polymicrobial fields. One record had both fields recorded as negative despite discordant raw ESBL/AmpC and MDR flags; its clinical data were retained, but those discordant phenotype flags were excluded from patient‐level phenotype numerators and denominators because they could not be linked to a recovered isolate.

Tubo‐ovarian abscess was diagnosed using clinical assessment together with imaging and procedural findings. Treatment procedures included laparoscopy, laparotomy, or drainage. Culture‐positive patients were those with at least one organism isolated from abscess material obtained during surgery or drainage. Culture‐negative patients had no organism isolated despite clinical and procedural evidence of tubo‐ovarian abscess.

### 2.3 Clinical Pathway, Empirical Antibiotics, and Procedural Decision‐Making

Because the study was retrospective, patients were not assigned to groups by protocol or randomization. Conservative intravenous antimicrobial therapy was generally attempted in clinically stable patients. Operative or drainage intervention was considered when there was suspected rupture, peritonitis, sepsis, persistent fever, persistent or worsening abdominal pain, rising or non‐improving WBC count or CRP after approximately 48–72 h of intravenous therapy, radiological enlargement of the abscess, or imaging features suggesting impending rupture or a complex abscess unlikely to respond to medication alone.

Imaging reassessment was based on clinical need rather than a universal scheduled imaging protocol. Ultrasonography and computed tomography were repeated when the treating team documented persistent fever, worsening pain, increasing inflammatory markers, diagnostic uncertainty, or concern for progression or rupture. Because reassessment was clinically driven rather than scheduled, differential imaging frequency may have influenced ascertainment of treatment failure.

Empirical intravenous antibiotic therapy was not identical for all patients. The regimens recorded in the medical records were ceftriaxone plus metronidazole plus doxycycline in 33 patients (50.0%), imipenem‐cilastatin plus amikacin in 26 patients (39.4%), ertapenem in 5 patients (7.6%), and piperacillin‐tazobactam in 2 patients (3.0%). These regimens reflected local clinical practice and patient severity rather than a prespecified comparative treatment protocol. Therefore, antibiotic regimen was described as an exposure characteristic and was not interpreted as a randomized treatment assignment. The timing of the first antibiotic dose relative to specimen collection was unavailable; therefore, pre‐culture antibiotic exposure could not be quantified.

### 2.4 Variables and Definitions

Baseline variables included age, residence, occupation, medical comorbidities, history of abdominal surgery, obstetric and gynecologic history, abdominal pain, fever, abdominal guarding, diarrhea, abdominal distension, abnormal vaginal discharge, ultrasound findings, computed tomography findings, number of abscesses, largest abscess diameter, preoperative WBC count, preoperative CRP concentration, operative approach, empirical antibiotic regimen, culture result, organism identification, and antimicrobial susceptibility profile. CRP was summarized as a continuous variable using the median and minimum–maximum range; no prognostic CRP threshold was used in the analysis. Age was analyzed as recorded in the source dataset; patient‐level admission dates were unavailable, so age at admission could not be independently recalculated.

The available microbiology data recorded a multidrug‐resistance phenotype using resistance across at least three antimicrobial classes. The international interim definition proposed by Magiorakos et al. defines MDR as acquired non‐susceptibility to at least one agent in three or more antimicrobial categories [14]. However, routine AST was not performed for all or nearly all relevant agents or categories for every isolate, and the archived dataset does not permit complete retrospective category‐level harmonization. Accordingly, MDR is reported as a “recorded MDR phenotype” rather than a standardized Magiorakos MDR prevalence. Intrinsic resistance was not interpreted as acquired resistance.

### 2.5 Microbiological Analysis

Purulent fluid or abscess material obtained during surgery or drainage was processed by the hospital microbiology laboratory. The reported microbiological spectrum therefore represents organisms recovered by routine clinical culture methods capable of recovering aerobic and facultatively anaerobic bacteria; dedicated obligate anaerobic culture was not performed. Organism identification and AST were performed according to routine hospital microbiology procedures. Instrument‐level identification‐platform metadata and isolate‐level AST technique were unavailable in the archived records. AST interpretation was based on the contemporaneous CLSI standards used by the hospital microbiology laboratory. The specific CLSI edition applied to each isolate could not be reconstructed retrospectively; M100 is cited as the general methodological standard [15]. Retrospective reinterpretation of all minimum inhibitory concentration data using a single CLSI version was not possible. Antibiotic‐specific denominators are therefore reported in Table 3.

Anaerobic culture and molecular testing for *Chlamydia trachomatis*, *Neisseria gonorrhoeae*, and other difficult‐to‐culture organisms were not available in the source dataset. Consequently, absence of anaerobes or sexually transmitted pathogens should be interpreted as a laboratory limitation rather than evidence that these organisms were absent. The culture results should not be used to justify discontinuation of anaerobic coverage when clinical features support tubo‐ovarian abscess.

### 2.6 Statistical Analysis

Data were entered using Microsoft Excel 2010 and analyzed using Stata 14 (StataCorp LLC, College Station, TX, USA). Categorical findings were summarized as counts and percentages. For clinical symptoms, percentages describe the proportion of the full 66‐patient sample in whom the finding was documented as present; these are not prevalence estimates among records with nonmissing symptom documentation, and the number with missing or unrecorded information is reported separately. Laboratory summaries used available measurements without imputation; the denominator is stated when values were missing. For AST, the denominator was the number of isolates tested for each antibiotic. Age was summarized using mean ± standard deviation, whereas WBC count, CRP concentration, and maximum abscess diameter were summarized using the median and minimum–maximum range; no formal normality test was used. Wilson 95% confidence intervals were calculated for selected key proportions. Analyses addressing the microbiological objective were descriptive; no null‐hypothesis significance tests or P values are reported, and no a priori alpha level or one‐ versus two‐sided testing specification was applicable. Statistical reporting was reviewed against the recommendations of Assel et al. and the SAMPL guidance [16, 17].

Analyses were restricted to descriptive summaries because the microbiological objective, incomplete isolate‐level linkage and AST panels, and limited event counts did not support stable confirmatory modeling. No imputation, receiver‐operating characteristic cut‐off derivation, or multivariable prediction model was performed. This case series was cross‐checked against the PROCESS 2023 guideline [12] and the STROBE checklist for observational studies [13].

### 2.7 Ethical Considerations

The study protocol was approved by the Biomedical Research Ethics Committee of Nhan Dan Gia Dinh Hospital (Certificate No. 29/NDGD‐HDDD; approval date: February 28, 2025) through an expedited review procedure. The approval certificate identifies the approved research period as October 2024–October 2025. The retrospective analysis included clinical records from January 2021 through May 2025. The requirement for informed consent was waived by the Biomedical Research Ethics Committee for this retrospective medical‐record study. The analytical dataset was de‐identified before analysis, with direct patient identifiers removed.

## 3 Results

### 3.1 Study Sample and Baseline Characteristics

The analytic sample comprised 66 records of surgically treated or drained tubo‐ovarian abscesses with procedure‐obtained abscess material submitted for culture. Antimicrobial susceptibility data were summarized for recovered isolates when available.

The mean age was 44.6 ± 10.2 years. Most patients resided in Ho Chi Minh City (51/66, 77.3%). Diabetes mellitus was documented in 9 patients (13.6%), and a history of previous abdominal surgery was documented in 27 (40.9%).

Abdominal pain was documented as present in 52/66 patients (78.8%), with 11/66 (16.7%) records lacking documentation of this symptom. Fever was documented as present in 37/66 (56.1%), with 11/66 (16.7%) unrecorded; abdominal guarding was documented as present in 21/66 (31.8%), with 13/66 (19.7%) unrecorded. These percentages use the full analytic sample as the denominator and should not be interpreted as prevalence among records with nonmissing symptom documentation. Preoperative CRP was available for 61/66 patients, with 5/66 (7.6%) missing. The median preoperative WBC count was 17 × 10^9^/L (minimum–maximum, 6–38), the median preoperative CRP concentration among 61 available measurements was 143 mg/L (range, 38–480), and the median maximum abscess diameter was 7.0 cm (range, 3.0–18.0). Baseline characteristics are summarized in Table 1.

**Table 1:** Baseline clinical, laboratory, imaging, procedural, and empirical antibiotic characteristics of the analytic sample (*N* = 66).

Characteristic	Summary type	Value
Age, years	Mean ± SD	44.6 ± 10.2
Residence in Ho Chi Minh City	*n*/*N* (%)	51/66 (77.3)
Diabetes mellitus	*n*/*N* (%)	9/66 (13.6)
Previous abdominal surgery	*n*/*N* (%)	27/66 (40.9)
Abdominal pain	Documented present, *n*/66 (%); missing separately	52/66 (78.8); missing 11/66 (16.7)
Fever	Documented present, *n*/66 (%); missing separately	37/66 (56.1); missing 11/66 (16.7)
Abdominal guarding	Documented present, *n*/66 (%); missing separately	21/66 (31.8); missing 13/66 (19.7)
Preoperative WBC count, ×10^9^/L	Median (minimum–maximum)	17 (6–38)
Preoperative CRP, mg/L (*n* = 61)	Median (minimum–maximum)	143 (38–480)
Maximum abscess diameter, cm	Median (minimum–maximum)	7.0 (3.0–18.0)
One abscess	*n*/*N* (%)	53/66 (80.3)
Two abscesses	*n*/*N* (%)	13/66 (19.7)
Laparoscopy	*n*/*N* (%)	50/66 (75.8)
Laparotomy	*n*/*N* (%)	13/66 (19.7)
Drainage	*n*/*N* (%)	3/66 (4.5)
Ceftriaxone + metronidazole + doxycycline	*n*/*N* (%)	33/66 (50.0)
Imipenem‐cilastatin + amikacin	*n*/*N* (%)	26/66 (39.4)
Ertapenem	n/*N* (%)	5/66 (7.6)
Piperacillin‐tazobactam	*n*/*N* (%)	2/66 (3.0)

### 3.2 Imaging and Empirical Antibiotic Patterns

Ultrasonography identified tubo‐ovarian abscess in 36 patients (54.6%), while computed tomography identified tubo‐ovarian abscess in 41 patients (62.1%). Computed tomography was not performed in 10 patients (15.2%). A single abscess was recorded in 53 patients (80.3%), and two abscesses were recorded in 13 patients (19.7%).

The most frequently documented empirical regimen was ceftriaxone plus metronidazole plus doxycycline (33/66, 50.0%). Broader Gram‐negative regimens were also common: imipenem‐cilastatin plus amikacin was used in 26 patients (39.4%), ertapenem in 5 (7.6%), and piperacillin‐tazobactam in 2 (3.0%).

### 3.3 Culture Yield and Microbiological Spectrum

Culture positivity was 43.9% (29/66; 95% CI, 32.6%–55.9%), with 37/66 (56.1%) culture‐negative patients. Among the 29 culture‐positive patients, 23 (79.3%) were classified as monomicrobial and 6 (20.7%) as polymicrobial based on the mono‐/polymicrobial fields in the analytic dataset. The available microbiology data reported 39 bacterial isolates from these 29 patients, confirming that more than one isolate was recovered from at least some patients; however, an isolate‐level patient linkage file was not available to map all 39 isolates to individual patients. Escherichia coli was the leading isolate (19/39, 48.7%), followed by Streptococcus spp. (9/39, 23.1%) and Klebsiella pneumoniae (7/39, 17.9%).

Among the 19 E. coli isolates, 6 (31.6%) were extended‐spectrum beta‐lactamase producers and 1 (5.3%) was an AmpC producer. Therefore, 7 of 19 E. coli isolates (36.8%) had an ESBL or AmpC phenotype. No ESBL and AmpC co‐producing *E. coli* isolate was recorded in the available microbiology data. The complete microbiological distribution is presented in Table 2.

**Table 2:** Microbiological spectrum and recorded multidrug‐resistance phenotype of tubo‐ovarian abscesses managed by surgery or drainage.

Variable or organism	Numerator/Denominator	Percentage
Culture‐positive patients	29/66	43.9
Culture‐negative patients	37/66	56.1
Monomicrobial among culture‐positive patients	23/29	79.3
Polymicrobial among culture‐positive patients	6/29	20.7
Total bacterial isolates	39	100.0
*Escherichia coli*, total	19/39	48.7
ESBL‐producing *E. coli*	6/19	31.6
AmpC‐producing *E. coli*	1/19	5.3
ESBL/AmpC phenotype among *E. coli*	7/19	36.8
*Klebsiella pneumoniae*	7/39	17.9
*Streptococcus* spp., total	9/39	23.1
*Proteus mirabilis*	1/39	2.6
*Enterobacter cloacae*	1/39	2.6
*Stenotrophomonas maltophilia*	1/39	2.6
*Enterococcus faecium*	1/39	2.6
Isolates with recorded MDR phenotype	19/39	48.7

*Note:* ESBL and AmpC percentages were calculated among *E. coli* isolates. Percentages for bacterial species were calculated among all 39 reported isolates. Among the 29 culture‐positive patients, 23 were monomicrobial and 6 polymicrobial. The available microbiology data reported 39 isolates from these 29 patients, but an isolate‐level patient linkage file was not available to map all isolates to individual patients. MDR is reported as a recorded isolate phenotype rather than a strictly standardized Magiorakos MDR classification because routine AST panels were incomplete and varied across isolates. The available microbiology data do not support a reliable patient‐level MDR denominator among culture‐positive patients.

Abbreviations: ESBL, extended‐spectrum beta‐lactamase; MDR, multidrug resistance.

### 3.4 Antimicrobial Susceptibility Patterns

Among tested *E. coli* isolates, resistance was high to ampicillin (13/14, 92.9%), ampicillin‐sulbactam (7/14, 50.0%), ceftriaxone (7/14, 50.0%), and trimethoprim‐sulfamethoxazole (10/19, 52.6%). For levofloxacin, only 7 *E. coli* isolates had susceptibility results, and resistance was observed in 3 of 7 isolates. Susceptibility appeared comparatively preserved to piperacillin‐tazobactam (12/14, 85.7%), ertapenem (14/14, 100.0%), imipenem (13/14, 92.9%), and gentamicin (12/14, 85.7%).

*Klebsiella pneumoniae* isolates showed intrinsic non‐susceptibility to ampicillin in the tested isolates (5/5), which should not be interpreted as acquired resistance. The small number of *K. pneumoniae* isolates precluded reliable percentage‐based interpretation; therefore, these findings are reported as raw fractions in Table 3. Antibiotic‐specific susceptibility estimates should be interpreted cautiously because tested isolate numbers were small and varied across antimicrobial agents.

**Table 3:** Antimicrobial susceptibility patterns of *Escherichia coli* and *Klebsiella pneumoniae* isolates.

Antibiotic	*E. coli*	*E. coli*	*E. coli*	*K. pneumoniae*	*K. pneumoniae*	*K. pneumoniae*
	**S**	**I**	**R**	**S**	**I**	**R**
Ampicillin*	1/14 (7.1)	0/14 (0.0)	13/14 (92.9)	0/5	0/5	5/5
Ampicillin‐sulbactam	7/14 (50.0)	0/14 (0.0)	7/14 (50.0)	5/6	0/6	1/6
Piperacillin‐tazobactam	12/14 (85.7)	0/14 (0.0)	2/14 (14.3)	5/5	0/5	0/5
Ceftriaxone	7/14 (50.0)	0/14 (0.0)	7/14 (50.0)	6/6	0/6	0/6
Cefepime	8/12 (66.7)	0/12 (0.0)	4/12 (33.3)	5/5	0/5	0/5
Ertapenem	14/14 (100.0)	0/14 (0.0)	0/14 (0.0)	6/6	0/6	0/6
Imipenem	13/14 (92.9)	1/14 (7.1)	0/14 (0.0)	6/6	0/6	0/6
Gentamicin	12/14 (85.7)	1/14 (7.1)	1/14 (7.1)	5/5	0/5	0/5
Levofloxacin	3/7	1/7	3/7	2/3	0/3	1/3
Trimethoprim‐sulfamethoxazole	9/19 (47.4)	0/19 (0.0)	10/19 (52.6)	5/7	0/7	2/7

*Note:* Values are n/N; percentages are shown only when the antibiotic‐specific denominator was at least 10. Raw fractions are shown for denominators below 10 to avoid false precision. S, susceptible; I, intermediate; R, resistant. Intermediate isolates were not grouped with resistant isolates unless explicitly stated. The asterisk denotes intrinsic ampicillin non‐susceptibility in *K. pneumoniae*, which was not interpreted as acquired resistance. Because isolate numbers were limited and antibiotic‐specific denominators varied, susceptibility estimates should be interpreted cautiously.

### 3.5 Multidrug Resistance: Descriptive Summary

The available microbiology data classified 19 of 39 isolates (48.7%) as having a recorded multidrug‐resistance phenotype. Because isolate‐level AST panels were incomplete and varied by organism and antibiotic, this proportion should not be interpreted as a strictly standardized Magiorakos MDR prevalence. The available microbiology data do not provide sufficiently reliable patient‐isolate linkage to derive a patient‐level MDR proportion among the 29 culture‐positive patients. The recorded MDR phenotype is summarized together with the microbiological spectrum in Table 2.

## 4 Discussion

This retrospective case series provides local descriptive data on the microbiological spectrum and antimicrobial susceptibility patterns of surgically treated or drained tubo‐ovarian abscesses in a Vietnamese tertiary hospital. Culture positivity was modest, enteric Gram‐negative organisms and streptococci were frequently recovered, and *E. coli* showed substantial resistance to several commonly used agents. These findings apply specifically to a procedure‐treated, culture‐sampled population and should not be generalized to all patients with tubo‐ovarian abscess.

The microbial distribution is clinically plausible because tubo‐ovarian abscess can involve genital‐tract flora, enteric Gram‐negative organisms, streptococci, sexually transmitted pathogens, and obligate anaerobes [1, 7, 8, 11]. The absence of *Bacteroides fragilis*, *Peptostreptococcus*, and other obligate anaerobes in this series should not be interpreted as their true absence from the abscess cavity. Dedicated anaerobic culture was not performed. This limitation is clinically important: the absence of recovered anaerobes cannot be used to justify omitting anaerobic activity from empirical TOA therapy. Empirical regimens should continue to include guideline‐recommended anaerobic coverage [1, 4]. Prior antimicrobial exposure was a plausible unmeasured contributor to culture‐negative results, but its effect on culture yield could not be quantified in this dataset [7].

The resistance profile has implications for local antimicrobial stewardship. Ceftriaxone plus metronidazole plus doxycycline was the most frequently documented empirical regimen, consistent with broad pelvic‐infection coverage [1, 4]. However, ceftriaxone resistance and ESBL/AmpC phenotypes among recovered *E. coli* isolates indicate that a third‐generation cephalosporin may not provide reliable Gram‐negative activity in every patient in this setting. Numerically higher in vitro susceptibility to piperacillin‐tazobactam, carbapenems, and gentamicin was based on small, antibiotic‐specific denominators and should not be interpreted as a basis for routine escalation. Local antibiograms, culture‐directed treatment, and stewardship remain preferable to automatic broad‐spectrum use [7, 8, 9, 10].

Direct contemporary Southeast Asian TOA‐specific resistance data remain limited, and comparisons with broader regional surveillance should be interpreted cautiously because infection syndromes, specimen sources, case mix, and AST methods differ. Nonetheless, the 50.0% ceftriaxone resistance observed among the 14 tested *E. coli* isolates in this series is similar in magnitude to the pooled 49.3% (95% CI, 37.3%–61.3%) ceftriaxone resistance reported for human *E. coli* isolates in a recent Southeast Asian meta‐analysis [18]. In a provincial hospital in Cambodia, ceftriaxone resistance was reported in 110/148 (74%) *E. coli* bloodstream isolates collected during 2020–2022 [19]. In Southern Thailand, a 2024 study of selected ESBL‐producing *E. coli* and *K. pneumoniae* clinical isolates reported universal ceftriaxone resistance and 64.3% resistance to trimethoprim‐sulfamethoxazole [20]. Because the Thai cohort was enriched for ESBL‐producing organisms, those rates are not directly comparable with TOA isolates that were not selected on the basis of ESBL production. Taken together, these regional data place our findings within a broader pattern of substantial Enterobacterales resistance while reinforcing the need for syndrome‐specific local antibiograms rather than direct transfer of resistance estimates between populations.

Treatment and procedural decisions were not governed by a predefined standardized protocol, and imaging reassessment was clinically driven. Published studies and recent meta‐analyses have associated larger abscess size, inflammatory burden, and selected clinical characteristics with failure of medical treatment or need for intervention, although reported predictors and thresholds vary across populations [21, 22, 23, 24, 25, 26, 27]. Image‐guided drainage has also been evaluated as an adjunct to antibiotics in contemporary series [24, 28]. These practice variations may have introduced selection bias toward patients with more severe, complicated, or treatment‐refractory disease in the present surgical case series.

Potential associations between inflammatory status and treatment response should be interpreted cautiously. Peker et al. evaluated NLR, PLR, SII, CRP, and abscess diameter in relation to medical treatment response; NLR, PLR, and SII showed limited predictive value, whereas CRP and abscess diameter differed between medical and surgical‐treatment groups [29]. Considered alongside recent treatment‐failure studies [23, 25, 27], these findings support future prospective work integrating clinical, inflammatory, radiological, and microbiological information rather than relying on a single marker.

The small sample, incomplete AST panels, and absence of a patient‐level microbiology linkage file preclude stable multivariable resistance prediction and precise patient‐level risk estimation.

The high culture‐negative fraction is another important finding. Culture negativity may reflect prior antibiotics, sampling limitations, lack of dedicated anaerobic culture, and absence of molecular diagnostics [7, 8, 11]. Prior antimicrobial exposure before procedure‐obtained sampling may have influenced culture recovery; however, its effect could not be quantified in this dataset [7].

Several selection mechanisms should be considered when interpreting these findings. The final analytic sample included only surgically treated or drained patients with procedure‐obtained culture data; therefore, the results do not represent all patients with tubo‐ovarian abscess or those who respond to conservative therapy. If missing culture data or procedure selection differed by disease severity, selection bias may have influenced the recovered microbiological spectrum. The recovered organisms may also partly reflect flora remaining cultivable after antimicrobial exposure.

Strengths of this study include a clearly defined surgical case series, extraction of culture and antimicrobial susceptibility data from real‐world clinical records, inclusion of empirical antibiotic patterns, and a conservative descriptive analytic approach. The study also provides local data from a Vietnamese tertiary hospital, where published tubo‐ovarian abscess microbiology and resistance data remain limited.

Other limitations are substantial. The study was retrospective and single‐center, with 66 included analytic records and only 29 culture‐positive patients. Among the 29 culture‐positive patients, 23 were classified as monomicrobial and 6 as polymicrobial; however, complete isolate‐to‐patient linkage for the 39 recovered isolates was unavailable. Routine AST panels were incomplete and differed across isolates, preventing strict retrospective harmonization to the Magiorakos MDR definition. One record contained discordant culture‐status and ESBL/MDR flags; because the phenotype flags could not be linked to a recovered isolate, they were excluded from patient‐level MDR estimation. Symptom documentation and CRP measurements were incomplete for some records and are reported explicitly as missing. Antibiotic regimens were not randomized, imaging reassessment was not standardized, anaerobic culture and molecular testing were unavailable, and archived laboratory records did not preserve sufficiently reliable instrument‐platform or isolate‐level AST‐method metadata for retrospective specification. These limitations preclude causal inference, generalized empirical antibiotic recommendations, and validated resistance prediction.

Although these data arise from a single Vietnamese tertiary hospital, the findings are relevant to other settings where prior antibiotic exposure, delayed culture results, and increasing Enterobacterales resistance complicate empirical treatment of pelvic infections. The study illustrates the importance of local microbiological surveillance rather than direct extrapolation from international pelvic inflammatory disease guidelines alone. For Vietnam and other low‐ and middle‐income settings, a practical next step is investment in clinical microbiology capacity, including reliable anaerobic culture, standardized specimen transport, and molecular panels when feasible, so that antimicrobial stewardship can be guided by local evidence rather than empirical escalation alone. Future work should prioritize multicenter prospective cohorts using harmonized case definitions and standardized specimen collection, transport, organism identification, and AST; incorporate dedicated anaerobic culture and molecular diagnostics for fastidious pathogens and resistance determinants; and preserve complete patient–isolate linkage. Such designs would permit more reliable estimates of syndrome‐specific resistance and prospective evaluation of culture‐guided treatment strategies.

## 5 Conclusion

In this single‐center retrospective case series of surgically treated or drained tubo‐ovarian abscesses, culture‐positive cases most frequently yielded *E. coli*, *Streptococcus* spp., and *K. pneumoniae*. Among tested *E. coli* isolates, resistance was frequent to ampicillin, ceftriaxone, and trimethoprim‐sulfamethoxazole, whereas susceptibility appeared higher to piperacillin‐tazobactam, carbapenems, and gentamicin; these estimates are limited by small and variable testing denominators. The findings support culture of procedure‐obtained abscess material, locally updated antibiograms, clinically indicated anaerobic coverage, and strengthened microbiology capacity. Larger multicenter prospective studies using standardized microbiological methods, complete AST panels and patient–isolate linkage, dedicated anaerobic culture, and molecular diagnostics are needed.

## Author Contributions

**Bui Chi Thuong:** conceptualization, methodology, supervision. **Huynh Tan An:** investigation, validation, data curation. **Pham Dinh Nguyen:** conceptualization, methodology, writing – review and editing, formal analysis, project administration, visualization, writing – original draft.

## Funding

The authors have nothing to report.

## Disclosure

All authors have read and approved the final version of the article. Pham Dinh Nguyen had full access to all the data in this study and takes complete responsibility for the integrity of the data and the accuracy of the data analysis.

## Ethics Statement

The study protocol was approved by the Biomedical Research Ethics Committee of Nhan Dan Gia Dinh Hospital (Certificate No. 29/NDGD‐HDDD; approval date: February 28, 2025) through an expedited review procedure. The approval certificate identifies the approved research period as October 2024–October 2025. The retrospective analysis included clinical records from January 2021 through May 2025. The requirement for informed consent was waived by the Biomedical Research Ethics Committee for this retrospective medical‐record study.

## Conflicts of Interest

The authors declare no conflicts of interest.

## Transparency Statement

The article guarantor, Pham Dinh Nguyen, affirms that this article is an honest, accurate, and transparent account of the study being reported; that no important aspects of the study have been omitted; and that any discrepancies from the study as planned have been explained.

## Supporting information


Supporting File 1

Supporting File 2

## Data Availability

The deidentified source data are not publicly available because they derive from clinical records and microbiology reports and remain subject to institutional, ethical, privacy, and confidentiality restrictions. Reasonable requests for access to deidentified data, the data‐collection materials, and information needed to reproduce the reported descriptive analyses may be directed to the corresponding author at nguyenpd@nhidong.org.vn and are subject to institutional and ethics approval. No standalone analytic code repository was created for this study; analyses were performed in Stata 14 as described in the article.
