# Supplementary material for: Microbiological Spectrum and Antimicrobial Resistance Patterns in Tubo‐Ovarian Abscesses Managed by Surgery or Drainage at a Tertiary Hospital in Vietnam: A Retrospective Case Series
Source: Health Sci Rep. 2026 Oct 26;9(11):e73414. doi: 10.1002/hsr2.73414 (PMC13700928; doi:10.1002/hsr2.73414)
Supplement: Supplementary file 1 [file HSR2-9-e73414-s001.docx]

**Supplementary Material S2
PROCESS 2023 Reporting Checklist Cross-Check**

**Study: Microbiological Spectrum and Antimicrobial Resistance Patterns in Tubo-Ovarian Abscesses Managed by Surgery or Drainage at a Tertiary Hospital in Vietnam: A Retrospective Case Series**

This checklist cross-check uses the PROCESS 2023 item structure and concise paraphrases of the reporting requirements. Items not supported by the retrospective analytic dataset are explicitly identified rather than inferred.

| **Item** | **Topic** | **PROCESS 2023 requirement (concise paraphrase)** | **How addressed in this manuscript** |
| --- | --- | --- | --- |
| 1 | Title | Identify the report as a case series and state its focus. | p. 1, lines 1–2. Reported in title: retrospective case series; TOA; surgery/drainage; tertiary hospital; Vietnam. |
| 2 | Keywords | Use 3–6 informative keywords including case series and surgical field. | p. 1, line 16; p. 4, lines 54–55. Six keywords include case series and gynecologic surgery. |
| 3a | Abstract | State background, rationale, theme, and objective. | p. 3, lines 32–35. Reported in Background and Aims. |
| 3b | Abstract | State sample size, timeframe, and key design characteristics. | p. 3, lines 36–41. Reported: N=66, January 2021–May 2025, single-center retrospective case series. |
| 3c | Abstract | Summarize principal outcomes/results. | p. 3, lines 42–48. Culture yield, organism distribution, ESBL/AmpC, AST findings, and MDR phenotype summarized. |
| 3d | Abstract | State key conclusion, clinical implications, and future direction. | pp. 3–4, lines 49–53. Reported in Conclusion with restricted generalizability and stewardship implications. |
| 3e | Abstract | Use a structured abstract appropriate to a formal case series. | pp. 3–4, lines 31–53. Structured under Background and Aims, Methods, Results, and Conclusion. |
| 4 | Highlights | Provide 3–5 concise key findings. | p. 4, lines 56–64. Three Key Points supplied as a separate file. |
| 5 | Introduction | Provide scientific background, rationale, theme, and objective. | pp. 4–5, lines 65–90. Reported in Introduction with contemporary TOA and AMR literature. |
| 6a | Registration | State research registration details, including retrospective registration when applicable. | pp. 5–6, lines 93–100. The manuscript states directly that the study was not registered before manuscript submission; therefore, no registration identifier or UIN is reported. |
| 6b | Protocol | State whether a study protocol was prepared, registered, or publicly accessible. | pp. 5–6, lines 93–100. The manuscript states directly that no separate publicly accessible protocol was available. |
| 6c | Ethical approval | State ethics requirement/body/approval details when applicable. | pp. 9–10, lines 189–196. Ethical approval body, certificate number, approval date, and consent waiver are reported. |
| 6d | Study design | State the case-series design and relevant design characteristics. | pp. 5–6, lines 91–100. Single-center retrospective case series stated in the title and Methods. Whether the 66 cases represented a consecutive or non-consecutive series and a larger sampling frame could not be reconstructed from the available records. |
| 6e | Setting and timeframe | Describe the clinical/geographic setting and relevant dates. | pp. 5–6, lines 93–100. Nhan Dan Gia Dinh Hospital, Ho Chi Minh City, Vietnam; retrospective record interval reported as January 2021–May 2025. |
| 6f | Participants | Describe participant characteristics, selection, eligibility, de-identification, and relevant outcomes. | p. 6, lines 101–115; pp. 9–10, lines 189–196. The analytic sample comprised 66 included records meeting the reported diagnosis/procedure/culture criteria. The analytical dataset was de-identified before analysis; a larger screening denominator was unavailable. |
| 6g | Recruitment | Describe recruitment/source of cases and incentives where relevant. | p. 6, lines 101–115. Cases were identified retrospectively from medical records; no prospective recruitment or participant incentives applied. |
| 6h | Pre-intervention optimization | Describe relevant pre-intervention optimization when available. | pp. 6–7, lines 116–135. Pre-procedure optimization was not systematically captured in the analytic dataset and is not presented as a comparative exposure. |
| 6i | Intervention | Describe intervention types, aims, and concurrent treatment. | pp. 6–7, lines 116–135; p. 11, lines 214–221. Laparoscopy, laparotomy, drainage, and empirical antimicrobial regimens are reported. |
| 6j | Intervention specifics | Describe relevant intervention rationale, timing, techniques, and treatment details. | pp. 6–7, lines 116–135. Clinical indications for intervention and empirical regimens are reported; granular operative-device details were not part of the study objective or analytic dataset. |
| 6k | Operator details | Describe operator training and experience where relevant. | pp. 6–7, lines 116–135. Operator-level training and experience were unavailable in the analytic dataset and therefore are not reported. |
| 6l | Quality control | Describe measures addressing procedural quality and variation where relevant. | pp. 6–7, lines 116–135; pp. 14–16, lines 287–327. This was a retrospective routine-care series; no study-specific procedural standardization was imposed. Treatment heterogeneity is acknowledged. |
| 6m | Postoperative care/follow-up | Describe postoperative care and follow-up when relevant to the study outcomes. | pp. 14–16, lines 287–327. The study objective concerned microbiology and AST rather than postoperative outcomes; systematic postoperative follow-up variables were not analyzed. |
| 6n | Analysis | Describe the statistical methods and analytical approach used for the case series. | p. 9, lines 170–188. Descriptive analyses used explicit numerators and denominators; continuous variables were summarized as prespecified; Wilson 95% confidence intervals were calculated for selected key proportions; no imputation, null-hypothesis testing, or multivariable prediction modeling was performed. |
| 7a | Results—participants | Report number and characteristics, preferably in a table. | pp. 10–11, lines 199–213; Table 1, pp. 22–23. N=66 and baseline characteristics reported in Results and Table 1, including missingness. |
| 7b | Results—deviations | Report deviations from planned management when relevant. | pp. 6–7, lines 116–135. No study-assigned management plan existed; care was routine and nonrandomized. |
| 7c | Results—outcomes/follow-up | Report outcomes and follow-up relevant to the study. | pp. 11–12, lines 223–244; Tables 2–3, pp. 23–25. Microbiological outcomes are reported. Longitudinal postoperative outcomes were outside the descriptive microbiology objective. |
| 7d | Results—intervention adherence | Report adherence/tolerance when relevant. | pp. 6–7, lines 116–135. Not applicable to a nonrandomized retrospective microbiology-focused case series. |
| 7e | Results—complications/adverse events | Report complications/adverse events when relevant. | pp. 10–12, lines 197–251. Not analyzed as a study endpoint; no complication estimates are presented. |
| 8a | Discussion—key results | Summarize key results with relevant raw data/tables. | pp. 12–13, lines 253–258; Tables 2–3, pp. 23–25. Culture yield, organism distribution, AST denominators, and recorded MDR phenotype summarized with Tables 2–3. |
| 8b | Discussion—context | Compare with relevant literature and discuss clinical implications. | pp. 12–16, lines 252–339. Discussion addresses TOA microbiology, the empirical-treatment implications of unavailable anaerobic culture, regional Southeast Asian AMR context, antimicrobial stewardship, and treatment literature. |
| 8c | Discussion—strengths | Describe study strengths and cross-disciplinary relevance. | pp. 12–16, lines 252–339. Strengths include explicit denominators, missingness reporting, conservative interpretation, and local gynecology/microbiology/stewardship relevance. |
| 8d | Discussion—limitations | Describe weaknesses and their likely impact. | pp. 14–16, lines 287–327. Single center, selected procedure-treated population, culture-recovery limitations, incomplete AST panels, missing platform/technique metadata, absent anaerobic/molecular testing, and incomplete isolate linkage are reported. |
| 8e | Discussion—future research | Identify implications for future research. | pp. 15–16, lines 328–348. Future research explicitly prioritizes multicenter prospective studies with harmonized case definitions, standardized specimen collection/transport and microbiological methods, complete AST and patient–isolate linkage, dedicated anaerobic culture, and molecular diagnostics. |
| 8f | Discussion—cost effectiveness | Address cost-effectiveness when relevant. | pp. 12–16, lines 252–339. Not assessed; no economic comparison was performed. |
| 9a | Patient perspective | Report patient perspective when collected. | pp. 9–10, lines 189–196. Not collected in this retrospective medical-record study. |
| 9b | Informed consent | Report consent status as applicable. | pp. 9–10, lines 189–196. Consent status is stated in the ethics section. |
| 9c | Additional information | Report relevant conflicts or contextual information. | pp. 17–18, lines 354–384. Funding, conflicts, data availability, transparency, and author declarations are provided. |
| 10 | Conclusion | Provide a concise evidence-based conclusion. | p. 16, lines 340–348. Reported; inference remains restricted to procedure-treated/culture-sampled patients and the need for multicenter prospective validation is stated. |
| 11 | Declarations | Report conflicts of interest. | p. 17, lines 372–373. No conflicts declared. |
| 12a | Funding | State funding source and role. | p. 17, lines 368–371. No specific funding; no funder role. |
| 12b | Guarantor | Identify guarantor/responsible author where applicable. | p. 18, lines 381–384. The Transparency Statement identifies Pham Dinh Nguyen as the manuscript guarantor. |
| 12c | Provenance/peer review | State provenance/peer-review information if requested by journal. | N/A: handled by the journal editorial process. |
| 13 | Clinical media | Include relevant images/video when available and appropriate. | pp. 10–12, lines 197–251. Not required for the microbiology/AST objective; no screening-flow figure is presented because hospital-wide screening flow could not be reconstructed. |
| 14 | Checklist citation | Cite PROCESS 2023 and state reporting alignment. | pp. 5–6, lines 93–100. Methods cite PROCESS 2023, state reporting alignment, and directly report the study registration and protocol status. |

**Reference:** Mathew G, Sohrabi C, Franchi T, Nicola M, Kerwan A, Agha R; PROCESS Group. Preferred Reporting Of CasE Series in Surgery (PROCESS) 2023 guidelines. Int J Surg. 2023;109(12):3760–3769. doi:10.1097/JS9.0000000000000940.
