# Supplementary material for: Microbiological Spectrum and Antimicrobial Resistance Patterns in Tubo‐Ovarian Abscesses Managed by Surgery or Drainage at a Tertiary Hospital in Vietnam: A Retrospective Case Series
Source: Health Sci Rep. 2026 Oct 26;9(11):e73414. doi: 10.1002/hsr2.73414 (PMC13700928; doi:10.1002/hsr2.73414)
Supplement: Supplementary file 2 [file HSR2-9-e73414-s002.docx]

**Supplementary Material S1
STROBE Checklist**

**Manuscript title: Microbiological Spectrum and Antimicrobial Resistance Patterns in Tubo-Ovarian Abscesses Managed by Surgery or Drainage at a Tertiary Hospital in Vietnam: A Retrospective Case Series**

All STROBE items and subitems are addressed below; items not applicable to this descriptive retrospective case series are explicitly identified.

| **Section** | **Item** | **Reporting item** | **Where/how addressed** |
| --- | --- | --- | --- |
| Title and abstract | 1a | Indicate the study design in the title or abstract. | p. 1, lines 1–2; pp. 3–4, lines 31–53. Title and Abstract—Methods explicitly identify a retrospective case series. |
| Title and abstract | 1b | Provide a balanced summary of methods and findings. | pp. 3–4, lines 31–53. Structured Abstract reports setting, sample, microbiology, AST findings, limitations, and conclusion. |
| Background/rationale | 2 | Explain scientific background and rationale. | pp. 4–5, lines 65–86. Introduction, paragraphs 1–3. |
| Objectives | 3 | State specific objectives and hypotheses if prespecified. | p. 5, lines 87–90. End of Introduction states descriptive microbiology and susceptibility objectives; no confirmatory hypothesis was prespecified. |
| Study design | 4 | Present key design elements early. | pp. 5–6, lines 91–100. Methods—Study Design and Setting. |
| Setting | 5 | Describe setting, location, and relevant dates. | pp. 5–6, lines 93–100. Nhan Dan Gia Dinh Hospital, Ho Chi Minh City; retrospective record interval reported for this analysis: January 2021–May 2025. |
| Participants | 6a | Describe eligibility criteria and participant selection. | p. 6, lines 101–115. Methods—Participants and Case Selection: procedure-treated/drained TOA and abscess material submitted for culture; a complete screening log and total-sampling status were unavailable. |
| Participants | 6b | For matched studies, report matching criteria and numbers. | N/A: no matching and no comparison group. |
| Variables | 7 | Define outcomes, exposures, predictors, confounders, and diagnostic criteria as applicable. | pp. 7–8, lines 136–152. Methods—Variables and Definitions; culture status, AST denominators, recorded MDR, clinical and imaging variables defined. |
| Data sources/measurement | 8 | Describe data sources and measurement methods. | pp. 7–9, lines 136–169. Methods—Variables and Definitions and Microbiological Analysis. Archived platform and isolate-level AST-method metadata were unavailable. |
| Bias | 9 | Describe efforts to address potential bias. | pp. 6–7, lines 116–135; p. 9, lines 170–188; pp. 14–16, lines 287–327. Methods and Discussion address selection, prior antibiotics, culture negativity, missingness, incomplete AST, and absent patient–isolate linkage. |
| Study size | 10 | Explain how study size was determined. | pp. 5–6, lines 91–115. The study included N=66 records. A larger sampling frame and screening denominator could not be reconstructed; no a priori sample-size calculation was used for this descriptive case series. |
| Quantitative variables | 11 | Explain handling of quantitative variables and reasons for groupings. | pp. 7–9, lines 136–188. Age is summarized as mean ± SD and was analyzed as recorded; patient-level admission dates were unavailable, so age at admission could not be independently recalculated. WBC, CRP, and maximum abscess diameter are summarized using median and minimum–maximum range. CRP is analyzed as a continuous descriptive variable; no prognostic threshold is used. |
| Statistical methods | 12a | Describe statistical methods, including confounding control if applicable. | p. 9, lines 170–188. Descriptive summaries and Wilson 95% CIs; no adjusted model, so confounding control was not applicable. |
| Statistical methods | 12b | Describe subgroup and interaction methods. | p. 9, lines 170–188. N/A: no inferential subgroup or interaction analyses. |
| Statistical methods | 12c | Explain handling of missing data. | p. 9, lines 170–188; pp. 10–11, lines 199–213; Table 1, pp. 22–23. No imputation; available-case denominators and missing symptom/CRP documentation are reported. |
| Statistical methods | 12d | Describe design-specific analytical handling if applicable. | p. 9, lines 170–188. N/A: no matching, longitudinal follow-up analysis, or complex-sampling analysis was performed. |
| Statistical methods | 12e | Describe sensitivity analyses. | p. 9, lines 170–188. None performed; descriptive objective and incomplete isolate-level data did not justify sensitivity modeling. |
| Participants | 13a | Report numbers at each study stage. | p. 6, lines 101–115; pp. 10–12, lines 197–251. Results report 66 included analytic records. A larger hospital-wide screening denominator could not be reconstructed from the available records. |
| Participants | 13b | Give reasons for non-participation or exclusion. | p. 6, lines 101–115. Inclusion criteria are reported. Pre-analytic exclusion reasons and counts could not be reconstructed because a complete screening log was unavailable. |
| Participants | 13c | Consider a flow diagram. | p. 6, lines 101–115. A flow diagram is not provided because a valid hospital-wide screening flow could not be reconstructed from the available records. |
| Descriptive data | 14a | Report participant characteristics and relevant clinical information. | pp. 10–11, lines 199–213; Table 1, pp. 22–23. Results—Baseline Characteristics; Table 1. |
| Descriptive data | 14b | Indicate numbers with missing data. | pp. 10–11, lines 205–213; Table 1, pp. 22–23; p. 12, lines 235–244; Table 3, pp. 24–25. Results/Table 1 report symptom and CRP missingness; AST uses tested-isolate denominators. |
| Descriptive data | 14c | For cohort studies, summarize follow-up time. | N/A: longitudinal follow-up was not an analyzed outcome. |
| Outcome data | 15 | Report outcome/event data appropriate to the design. | pp. 11–12, lines 223–251; Tables 2–3, pp. 23–25. Results—culture yield, organisms, AST, and recorded MDR phenotype. |
| Main results | 16a | Give unadjusted estimates and precision; adjusted estimates if applicable. | p. 9, lines 170–188; pp. 11–12, lines 223–244; Tables 2–3, pp. 23–25. Culture positivity includes a Wilson 95% CI; AST denominators are explicit. Adjusted estimates N/A. |
| Main results | 16b | Report category boundaries for categorized continuous variables. | pp. 7–8, lines 136–152; p. 12, lines 235–244; Table 3, pp. 24–25. No prognostic cut-points or risk categories were derived. Antibiotic-specific AST denominators and raw fractions are reported explicitly. |
| Main results | 16c | Translate relative risks into absolute risks if relevant. | N/A: no relative-risk or time-to-event estimates. |
| Other analyses | 17 | Report other analyses such as subgroup or sensitivity analyses. | p. 12, lines 245–251; Table 2, pp. 23–24; p. 9, lines 170–188. No inferential subgroup, interaction, or sensitivity analyses; MDR summarized descriptively. |
| Key results | 18 | Summarize key results relative to objectives. | pp. 12–13, lines 252–258. Discussion opening paragraph. |
| Limitations | 19 | Discuss limitations, bias, and imprecision. | pp. 14–16, lines 287–327. Discussion details culture-recovery limitations, retrospective design, selected procedural population, missingness, incomplete AST, absent anaerobic/molecular testing, platform metadata, and linkage limitations. |
| Interpretation | 20 | Provide cautious interpretation considering objectives and limitations. | pp. 12–16, lines 252–348. Discussion and Conclusion provide cautious clinical interpretation, regional AMR contextualization, and avoid causal or universal empirical-treatment claims. |
| Generalisability | 21 | Discuss external validity. | pp. 15–16, lines 306–339. Discussion restricts inference to the selected procedure-treated/culture-sampled population and emphasizes local rather than directly transferred regional susceptibility estimates. |
| Funding | 22 | State funding source and funder role. | p. 17, lines 368–371. Funding declaration: no specific funding and no funder role. |
